# Geographic inequalities and determinants of anaemia among preeclamptic women: a cross-sectional sample-based study in Bangladesh

**DOI:** 10.1186/s12889-024-18176-8

**Published:** 2024-06-20

**Authors:** Ahasan Ali, Jahirul Islam, Ratna Paul, Shahinur Parvin, Abu Taiub Mohammed Mohiuddin Chowdhury, Rafiqul Islam, Sharmina Siddique, Atiqur Rahman, Sayeda Tamanna Tasnim, Suraiya Hasna

**Affiliations:** 1 Department of Physiology and Pathophysiology, Xi’an Jiaotong University, Xi’an, Shaanxi China; 2 School of Public Health & Social Work, Queensland University of Technology, Brisbane, Australia; 3 Department of Gynecology and Obstetrics, Dhaka Medical College Hospital (DMCH), Dhaka, Bangladesh; 4 Department of Nursing, Xi’an Jiaotong University, Xi’an, Shaanxi P.R. China; 5 Department of Gastroenterology, First Affiliated Hospital of Xi’an Jiaotong University, Xi’an, P.R. China; 6 Daffodil International University Dhaka, Dhaka, Bangladesh; 7 Department of Obstetrics and Gynecology, Dhaka Medical College Hospital, Dhaka, Bangladesh; 8 Plastic, Aesthetic and Maxillofacial surgery, Xian Jiaotong University, Xi’an, Shaanxi P.R. China; 9 Department of Anthropology, Chittagong University, Dhaka, Bangladesh; 10 Ad-din Women Medical College Hospital Dhaka, Dhaka, Bangladesh

**Keywords:** Preeclampsia, Anaemia, Bangladesh

## Abstract

**Background:**

Anaemia among preeclamptic (PE) women is a major undefined health issue in Bangladesh. This study explored the risk factors associated with anaemia and mapped the regional influences to understand the geographical inequalities.

**Methods:**

Data from 180 respondents were prospectively collected from the Preeclampsia ward of Dhaka Medical College Hospital (DMCH), Bangladesh. Anaemia was defined as a blood haemoglobin level less than 11.0 g/dl. Preeclampsia was defined as systolic blood pressure (SBP) ≥ 140 mmHg and diastolic blood pressure (DBP) ≥ 90 mmHg with proteinuria. Factors associated with anaemia were explored using the chi-square test. Logistic regression (LR) was done to determine the level of association with the risk factors.

**Results:**

Among the participants, 28.9% were identified as having early onset and 71.1% reported late onset of PE. 38.9% of the subjects were non-anaemic, whereas mild, moderate, and severe anaemia was found among 38.3%, 17.8%, and 5% of patients respectively. The following factors were identified; including age range 25–34 (OR: 0.169, *p* < 0.05), a lower education level (OR: 3.106, *p* < 0.05), service-holder mothers (OR: 0.604, *p* < 0.05), pregnancy interval of less than 24 months (OR: 4.646, *p* < 0.05), and gestational diabetes mellitus (OR: 2.702, *p* < 0.05). Dhaka district (IR: 1.46), Narayanganj district (IR: 1.11), and Munshiganj district (IR: 0.96) had the highest incidence rates.

**Conclusion:**

Determinants of anaemia must be considered with importance. In the future, periodic follow-ups of anaemia should be scheduled with a health care program and prevent maternal fatality and fetus morbidity in patients with PE.

## Introduction

Preeclampsia (PE) is a new onset of hypertension (systolic blood pressure ≥ 140 mmHg and diastolic blood pressure ≥ 90 mmHg) with proteinuria after 20 weeks of gestation in previously normotensive women [1, 2]. According to the estimations, this disease affects 8–10% of the world’s population, and 20% of them live in underdeveloped nations [3]. To date, PE is one of the obstetric complications that are responsible for 70,000 maternal and 500,000 fetal deaths worldwide and 10% of maternal deaths in Asia [3, 4]. The mortality rate is 14 times greater in developing nations [5] and is not limited to any geographic zone [6–8]. The prevalence of PE in Bangladesh is 10% which is comparatively higher than in other Asian countries like China (2.07%), Japan (1.19%), Thailand (2.22%), and Nepal (0.59%) [9, 10]. The etiology of PE has not been fully elucidated [11] but anaemia is considered one of the known hazards [12].

Despite having no specific age, anaemia is a clinical illness that is more common in young children, pregnant women, and those in the reproductive stage (15–49) [13–15]. WHO defined anaemia as a haemoglobin level ˂ 11.0 g/dl for pregnant women and ˂ 12.0 g/dl for women who are not pregnant [16, 17]. Globally 1.62 billion people are anaemic, among them the most vulnerable group is pregnant women where a total of 56 million suffer from this complication [13, 18]. The prevalence of anaemia among pregnant women in Bangladesh is 42.2% which is quite high compared to the global prevalence of 40% [19, 20]. Anaemia is influenced by multiple reasons; the contributing factors may change based on the geographic situation [21]. Annual report of UNICEF demonstrated the variation of anaemia exists more in the rural areas (44.3%) compared to the urban (40.2%) [22]. However, over one-third of women who are of reproductive age experience this problem, and nearly 40% of them live in developing regions, such as sub-Saharan Africa [17, 23].. Based on the evidence, anaemia is influenced by multifactorial reasons such as seasonal influence, food habits, and geographical clusters [21, 24–26]. It inhibits oxygen transportation into the blood, resulting in negative effects of low birth weight (LBW) baby, preterm delivery, stillbirth, loss of productivity, fatigue, breathlessness, dizziness, and headaches, and even turns into fatal anaemia [27–31]. In 2000 and 2014, a significant number of premature births, fatal impairment, low birth weight as well as infant and more than half of maternal deaths were observed due to anaemia [32–35]. The prevalence of anaemia in 2019 among reproductive age women was 29.9% whereas it became 33.7% in 2021 [36]. Anaemia is a serious public health issue, with detrimental effects on a woman’s health [37]. Day by day its frequency is globally increased posing a concern for the 3rd world countries [31, 38]. Many studies have been conducted to find out the risk factors of anaemia in pregnant women but less data available among PE. The purpose of this study was to determine the important cause of anaemia in patients with PE as well as the geographic variations of anaemia.

## Methods

### Study settings

The Department of Gynaecology and Obstetrics at the Dhaka Medical College Hospital (DMCH) in Dhaka, Bangladesh, conducted a hospital-based cross-sectional study from September 2021 to August 2022. Ethical approval was obtained from the Institutional Review Board (IRB) of that medical college (ERC-DMC/ECC/2022/31). A total of 180 of the 210 preeclamptic pregnant mothers were recruited for the study after obtaining their consent. The patient’s anthropometric measurements, including height, weight, socio-demographic information, personal and family histories of diabetes, hypertension, and lifestyle preferences (working or sedentary), were recorded on a questionnaire form. Information about preexisting hypertension and the need for antihypertensive medications was acquired based on individual reports or medical records. Systolic blood pressure (SBP) and diastolic blood pressure (DBP) were measured at DMCH using mercury sphygmomanometer equipment after resting for at least 10 min. The participants’ blood pressure was checked from the left side, and they were weighed using a weighing scale (Beurer BF 700, Germany) without shoes and heavy clothing. The nearest 0.1 kg (kg) was used to measure weight.

### Inclusion criteria

Pregnant women with preeclampsia in the second trimester were recruited for this study. We defined this as women with previously normal blood pressure (BP) after the 20th week of gestation with two different measures of BP that were at least four hours apart, diastolic ≥ 90 mmHg and systolic ≥ 140 mmHg, with a dipstick value of 1 + proteinuria of 300 mg or more per 24-hour urine sample [39].

### Sampling

Exclusion and inclusion criteria were strictly followed during data and biological sample (blood) collection. We engaged an expert medical technologist (lab) to measure the maternal hemoglobin using spectrophotometry at DMCH.

### Sample size

By using this formula: n = z2q/r2p, we estimated the sample size. The following assumptions were considered: a significant level of 0.05 (1.96), a margin of error of 5%, and a proportion of anaemia counted at 30% in pregnant women. Another study depicted the prevalence of anaemia as 19–50% [40]. We contemplated 30%, and the sample size was 175, adding 10% non-response, so 192 was finalized. However, we were able to collect 180 samples due to resource constraints.

### Data collection tools and method

Face-to-face interviews were performed to assemble data using a previously tested (Ad-din Women Medical College Hospital, Dhaka, Bangladesh) semi-structured questionnaire. The questionnaire was written in English and translated into Bengali. Two BSc nurses were engaged in accumulating data and trained before data collection. The principal investigator coordinated data collection and checked the inclusiveness of the collected questionnaire. The medical record was reviewed for clinical investigation and another laboratory report.

### Dependent and independent variables

We defined the outcome variable (anaemia) in pregnant women by the WHO’s definition, where Hb < 11.0 g/dl counts as anaemic, 10.0–10.9 g/dl is mild, 7–7.9 g/dl is moderate, and < 7.0 g/dl is severe anaemia [17]. Independent variables, including age, mother’s education, profession, resident status, family members, family income, BMI, pregnancy interval, parity, use of a recreational substance, and use of contraceptive methods, are used in categories. Moreover, body mass index (BMI) was recommended as < 18.5 kg/m^2^ underweight, 18.5–23.9 kg/m^2^ normal, 24-27.9 kg/m^2^ overweight, and 28 kg/m^2^ obesity [41]. Gestational age was assessed by a gap between the last menstrual period (LMP) and the first day of being the expected mother. We classified the monthly (mon) household (HH) income (in Bangladeshi taka: BDT) of recruited study people’s families conferring to the World Bank’s (WB) Data Help Desk 2016 as follows: Low-income group: HH income of ≤ 6,946/mon; lower-mid income group: HH income: 6,947–27,336/mon; upper-mid-income group: HH income: 27,337–84,564/mon; high-income group: HH income of ≥ 84,564 BD/mon [42].

### Data analysis

All the information was accumulated in a Microsoft Excel spreadsheet, and then we analysed it using SPSS (Version 25.0). Descriptive statistics, the chi-square test, logistic regression, and area under the curve (AUC) were performed. Descriptive statistics were used for the demographic characteristics of all the variables included in the study. As our variables were categorical and the dependent variable had two categories (0 = normal, 1 = anaemia), we decided to conduct logistic regression, and before that, independent variables were shortlisted by performing a chi-square test. The independent variables were selected based on prior research and a conceptual framework. All the finally selected variables-age, education, mother’s profession, physical activity of the patient, parity of case, family member, the interval in pregnancy, and gestational diabetes mellitus-were input within the modelling, and the enter method was selected. *P* value < 0.05 was used to denote significance. To validate our model performance, we generated an AUC, and a ROC curve. We further conducted a mapping demonstration to indicate the incidence rate. ArcGIS Pro (Version 3.1.2) was used to formulate the map, and the following formula was used for the incidence rate:

Incidence rate (IR) = {Case (district-wise)} / Female population × 100,000.

The incidence rate was calculated for 4 categories: using the total number of anaemia cases, mild anaemia (10.0-10.9 g/dl), moderate anaemia (7.0-9.9 g/dl), and severe anaemia (< 7.0 g/dl).

## Results

### Socio-demographic characteristics

A total of 180 respondents attended this survey. 36.7% (*n* = 66) of the sample were aged 15 to 24 years, 55.0% (*n* = 99) were between the ages of 25 and 34 years, and only 8.3% (*n* = 15) were more than 35 years old. The mean age was 26.85 (SD ± 5.3). The mother’s education level was 62.2% (*n* = 112) below the secondary school certificate (SSC), and 37.8% (*n* = 68) passed the higher secondary school certificate (HSC) or above. 70.6% (*n* = 126) of the participants were housewives, and 29.4% (*n* = 53) were service members. According to family income, 70% (*n* = 126) of respondents had a lower mid-income, 19.4% (*n* = 35) had an upper mid-income, 9.4% (*n* = 17) had a low income, and 1.1% (*n* = 2) had a higher income. About 52.8% (*n* = 95) of study participants reported having a sedentary lifestyle, and 47.2% (*n* = 85) did exercise. 27.8% (*n* = 50) of respondents had more than five family members; in contrast, 72.2% (*n* = 130) had 1–4 family members. Regarding the use of recreational substances, 63.3% (*n* = 114) of the respondents reported not using any recreational substances, while 15.6% (*n* = 28) reported using only betel nuts (Table 1).

**Table 1:** Socio-demographic features of the respondents, *n* = 180

Characteristics	Frequency (%)
**Age**	
15–24	66 (36.7)
25–34	99 (55.0)
> 35	15 (8.3)
Mean ± SD	26.85 ± 5.3
**Living area**	
Urban	67 (37.2)
Rural	113 (62.8)
**Mother education**	
< SSC	112 (62.2)
HSC or above	68 (37.8)
**Mother profession**	
Service	53 (29.4)
Housewife	127 (70.6)
**Family income**	
low income (< 5360)	17 (9.4)
lower mid-income (5361–21,270)	126 (70.0)
upper mid-income (21,271–65,761)	35 (19.4)
High income (> 65,762)	02 (1.1)
**Physical activity of the patient**	
Exercise	85 (47.2)
No exercise	95 (52.8)
**Family member**	
1–4	130 (72.2)
> 5	50 (27.8)
**Use of the recreational substance**	
No	114 (63.3)
Betel nut	26 (14.4)
Nut	28 (15.6)
Tobacco leaf	08 (4.4)
Cigarette	04 (2.2)

The average SBP was 150.5 ± 12.4 mmHg and average DBP was 75.89 ± 5.7 mmHg among the respondents. In the parity of cases, 60.6% (*n* = 109) was 1–4 parity, 28.3% (*n* = 51) was prime gravida, and 10.5% (*n* = 11.1) was > 5. The majority of the participants, 37.2% (*n* = 67), used condoms as a contraceptive method, while others used natural barriers (33.2%, *n* = 60), pills (15%, *n* = 27), and implants (5%, *n* = 9). Interval in pregnancy, 82.2% (*n* = 148) had less than 24 months, and 17.8% (*n* = 32) had more than 24.1 months. A total of 28.9% samples reported an early onset of PE, while 71.1% reported a late onset of PE. Overall, 38.9% of participants had normal levels of haemoglobin, whereas 38.3% had mild anaemia, 5.0% had severe anaemia, and 17.8% had moderate anaemia. The obstetrical and anaemic characteristics of 180 respondents are illustrated in Table 2.

**Table 2:** Obstetrical and anaemic personality traits of the respondents, *n* = 180

Characteristics	Frequency (%)
**Blood pressure**	
SBP (mmHg)	150.47 ± 12.4
DBP (mmHg)	75.89 ± 5.7
**Parity of case**	
Primi gravida	51 (28.3)
1–4	109 (60.6)
> 5	20 (11.1)
**Contraceptive method**	
Condom	67 (37.2)
Natural barrier	60 (33.3)
OCP	04 (2.2)
Injection	08 (4.4)
Pill	27 (15.0)
Implant	09 (5.0)
Copper-T	05 (2.8)
**Interval in pregnancy**	
< 24 month	148 (82.2)
> 24.1 months	32 (17.8)
**BMI in pregnancy**	
Normal (18.5–23.9 kg/m^2^)	109 (60.4)
Overweight (24-27.9 kg/m^2^)	44 (25.4)
Obese (≥ 28 kg/m^2^)	04 (2.2)
Underweight (< 18.5 kg/m^2^)	23 (12.8)
**Gestational diabetics mellitus**	
Yes	52 (28.9)
No	128 (71.1)
**Gestational age**	
< 34 weeks	42 (23.3)
34–37 weeks	76 (42.2)
> 37 weeks	62 (34.4)
**Onset of PE**	
Early onset of PE	52 (28.9)
Late onset of PE	128 (71.1)
**Hb level in PE patient**	
Normal	70 (38.9)
Mild anaemia	69 (38.3)
Moderate anaemia	32 (17.8)
Severe anaemia	09 (5.0)

Note: SBP-systolic blood pressure, DBP-diastolic blood pressure, OCP- oral contraceptive pill.

**Fig. 1:**
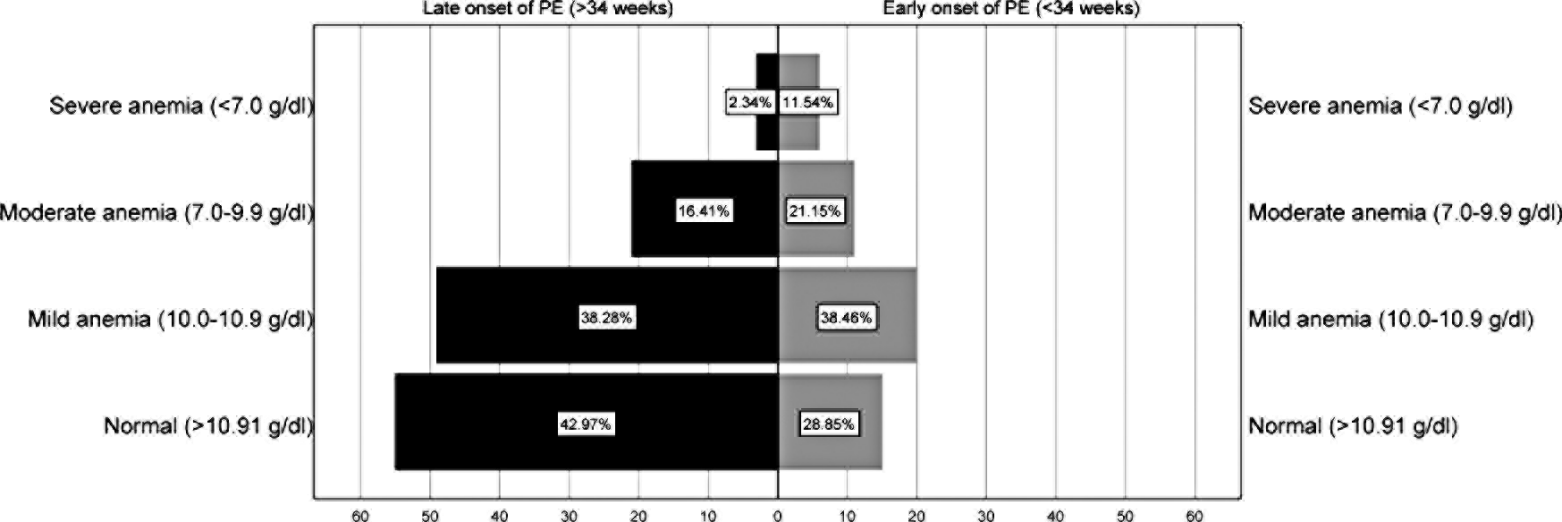
Prevalence of anaemia and outcome of PE among the respondents

The onset of PE was recorded into two categories, early onset (28.9%) and late onset (71.1%) of PE. The severe anaemia proportion was almost six times (11.54%) higher in the early onset of the PE group compared to late-onset (2.34%) PE. Overall, 42.97% of respondents had normal Hb when they delivered after 34 weeks of gestation while 28.85% had normal Hb before 34 weeks of delivery. Interestingly, the number of mild anaemias was nearly the same in both groups’ late onset of PE (38.28%) and early onset of PE (38.46%). However, moderate anaemia was comparatively lower (16.41%) in late-onset PE than (21.15%) in early-onset PE, as demonstrated in Fig. 1.

### Risk factors associated with anaemia

After performing a chi-square test, we found several indicators, including age, education, mother’s profession, physical activity, parity of case, number of family members, the interval in pregnancy, and GDM, which were significantly (*p* < 0.05) associated with anaemia. Then we conducted logistic regression to explore the level of association. We found that individuals in the “25–34” age category had statistically significantly lower odds (OR: 0.169; CI: 0.032–0.886) of the anaemia compared to individuals in the “> 35” age group. Individuals with “< SSC” education have statistically significantly higher odds (OR: 3.106; CI: 1.448–6.665) compared to individuals with higher education than SSC level. Mothers in the “service” profession have statistically significantly lower odds (OR: 0.604; CI: 0.263–1.388) compared to mothers who were “housewives.” Patients who were engaged in “exercise” have statistically significantly lower odds (OR: 0.414; CI: 0.188–0.913) compared to patients with “no exercise.” The outcome was not statistically significant for either ‘parity of case’ or ‘family member’. Individuals with an “interval in pregnancy” of “< 24 months” have statistically significantly higher odds (OR: 4.646; CI: 1.694–12.741) of anaemia compared to those with an “interval in pregnancy” of “>24.1 months.” Individuals with “gestational diabetes mellitus (GDM)” have significantly higher odds (OR: 2.702; CI: 1.172–6.228) compared to those who have no GDM. Table 3 describes the association between predictors and anaemia. We validated our model by checking the Akaike information criteria (AIC), and we found an AIC value of 0.794, indicating strong modelling performance.

**Table 3:** Association between predictor determinants and anaemia (Logistic Regression adjusted)

Variables	Category	n	RR (95% CI)	p. value
Age	15–24	66	0.297 (0.051-1.726)	> 0.05
	25–34	99	0.169 (0.032-0.886)	< 0.05
	> 35	15	1	
Education	< SSC	112	3.106 (1.448–6.665)	< 0.05
	> SSC	68	1	
Mother professor	Service	53	0.604 (0.263-1.388)	< 0.05
	Housewife	127	1	
Physical activity of the patient	Exercise	85	0.414 (0.188-0.913)	< 0.05
	No exercise	95	1	
Parity of case	Primi Gravida	55	0.536 (0.100 − 2.880)	> 0.05
	1–4	105	0.259 (0.057-1.190)	> 0.05
	> 5	20	1	
Family member	1–4	130	0.503 (0.199-1.269)	> 0.05
	> 5	50	1	
Interval of pregnancy	< 24 months	148	4.646 (1.694–12.741)	< 0.05)
	> 24.1 months	32	1	
GDM	Yes	52	2.702 (1.172–6.228)	< 0.05
	No	128	1	

### Geographical heterogeneity of the incidence rate (IR)

**Fig. 2:**
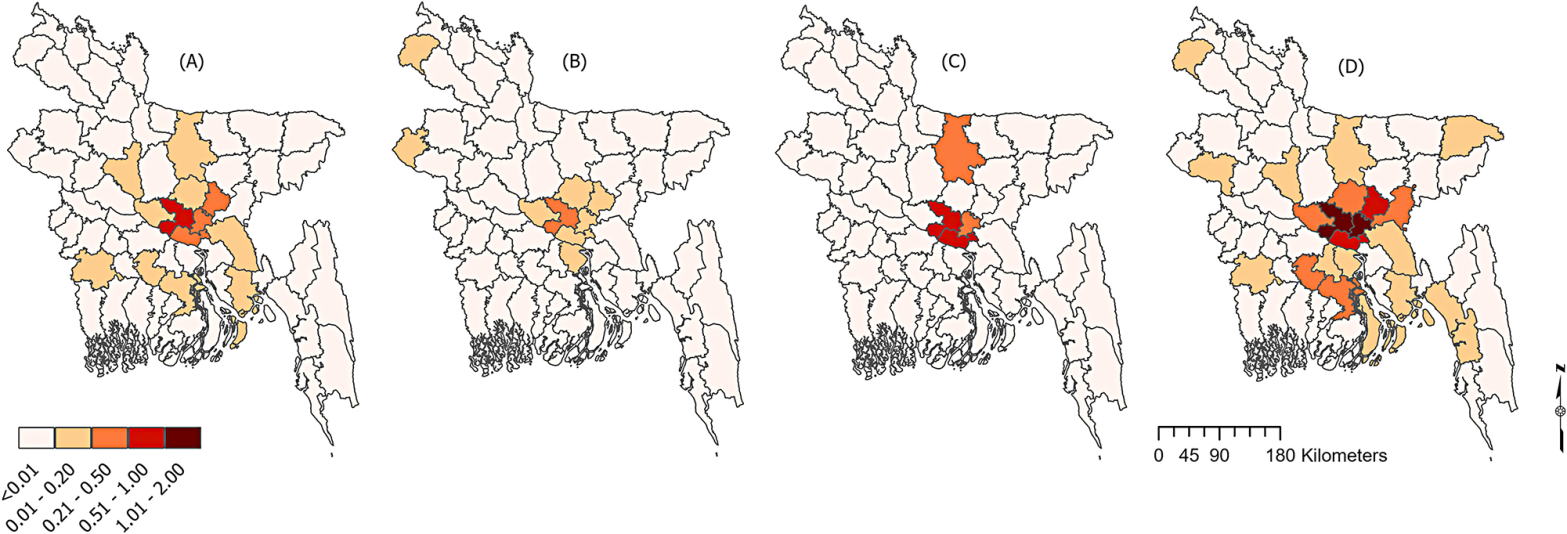
IR of the (**A**) Total number of anaemia cases (**B**) mild anaemia cases, (**C**) moderate anaemia cases, and (**D**) severe anaemia case

We divided the total anaemia cases based on the WHO definition of anaemia like mild, moderate, and severe anaemia. Most importantly, we further mapped the incidence rate and found the highest IR (mean severe anaemia rate) in Dhaka (IR: 1.46), Narayanganj (IR: 1.11), and Munshiganj (IR: 0.96) depicted in Fig. 2 (A). Interestingly, the higher IR of mild anaemia also showed the same places, such as Dhaka (IR: 0.54), Narayanganj (IR: 0.42), and Munshiganj (IR: 0.36). For the other types, we have illustrated those in Fig. 2, where the maximum IR was concentrated in the Dhaka district.

## Discussion

We focused on a capacity-based cross-sectional study to evaluate the determinants of maternal anaemia among preeclamptic women in Bangladesh. Anaemia is one of the major public health issues in pregnancy, affecting children’s and mothers’ health. In this study, we observed a high number (61.1%) of anaemia (Table 2), which was apex compared to the global prevalence (40.1%) [43]. This finding was comparable to other studies conducted in Bangladesh (59%), Bhutan (59%), and Sri Lanka (60%) [44], China (58.6%) [45], Malaysia (57.4%) [46], Kenya (57%), and Boditti Health Center (60%) [47, 48]. On the other hand, it was incompatible with some studies where anaemia prevalence was 16.6, 21.3, and 19%, respectively [49–51]. This discrepancy may be due to differences in methodology, like study period, sampling techniques, antenatal care, and iron supplementation during pregnancy. It might be an outcome of the gap between these studies and health service improvement. In the current study, pre-eclampsia and a single institution-based study might be the potential factors behind this result. Moreover, socioeconomic status in densely populous countries is a big obstacle of getting proper treatment in time. The present study illustrated that around 70% of respondents maintain their lives at a lower mid-income level (5361–21,270). Low economic and nutritional status may trigger a lower maternal haemoglobin level and preeclampsia in developing countries [52]. Our finding suggested that maternal age is associated with anaemia which is supported by several other studies [47, 53]. Maternal education (< SSC) was also significantly correlated with anaemia. This finding was compatible with the report of Erlindawati et al. Here, the literacy of the mother might hurt the attentiveness of antenatal care and health care services in the population [54]. In the present study, the maternal profession was connected to anaemia. This finding was inversed by other studies conducted in Gamo Gofa Zone, Ethiopia [55], Nepal [56], India [57], and in Walayta Soddo, Otona Hospital [58]. This may be careless of personal health due to a shortage of time that led to an adequate lack of personal hygiene, which exposed them to different types of parasitic infections. In the current study, anaemia prevalence was seen to rise with an adjacent interval. Study respondents who experienced a close pregnancy interval of less than 2 years were 4.64 times at increased risk of anaemia compared to those who had a more than 2 years birth interval. This result carried out a consistent trend with other studies conducted in Arba Minch [55], Bangladesh [44], Mogadishu [57], and Walayta Soddo [58]. These consequences might be connected to low iron storage in women’s bodies due to the rapid pregnancy succession between the following pregnancies. However, in Trinidad and Tobago performed another study and mentioned there was a relationship between pregnancy interval and the prevalence of anaemia [59]. In the above study, small sample size as well as methodological variation could be one of the reasons. The relationship between anaemia and GDM has not been fully documented, but in our current study, we found a strong association between GDM and anaemia with a low prevalence of GDM (28.9%). Similar findings have been reported by Lao et al. [60]. Our study observed that the majority of the cases are concentrated in the Dhaka district and Narayanganj district. Geographical clusters may provide a better illustration of the high-risk zone, which may assist in taking necessary actions in the future. Therefore, finding the geographical clusters opens up a future research scope. Another important issue is to check whether any climate parameter has an association with higher anaemia prevalence within the regions. Bangladesh is prone to vector-borne diseases like dengue, malaria, and chikungunya. While malaria and being anaemic are found to be associated, more study within the vector-borne endemic zones is required [61]. Due to climate change, several high-altitude regions, coastal areas, and deserts could be affected by dengue in the future. While seasonality is highly related to mosquito-borne diseases, numerous studies suggest that the seasonality of anaemia changes with malaria transmission [62]. Several authors further mentioned the high level of anaemia to nutritional deficiencies before and during the rainy season [63, 64]. This was supported by reports that heavy agricultural work in the rainy season aggravates anaemia and significantly reduces women’s weight [65, 66]. In south or central Asia, anaemia is significantly prevalent during monsoonal precipitation and at lower temperatures [65]. Therefore, future research in Bangladesh can focus on quantifying the association of climatic factors with anaemia prevalence. By using robust spatiotemporal models, the high-risk regions may be further differentiated to help with policy implementation.

Our study has several strengths, including the number of parameters we used to understand the sociodemographic characteristics of anaemia in Bangladesh. We further explored the association of the predictor variables to illustrate the major contributing factors associated with anaemia and mapped the disease incidence rate among the 64 districts in Bangladesh. Additionally, we used the AIC to check the validation and model performance. However, our study has some caveats. Along with the smaller sample size, we missed some other socio-economic and environmental predictors to check the association. Due to this reason, we were unable to explore the regional clusters, which may be addressed in future studies.

## Conclusion

Our findings indicated that multiple factors affect anaemia, and most importantly, anaemia is a factor that triggers preeclampsia. Bangladesh could face an increase in maternal mortality and morbidity in the future due to these two complex pathological conditions. This finding can be beneficial to policymakers in implementing programs to raise awareness about maternal health.

## Data Availability

The data set used and/or analysed during the current study is available from the corresponding author upon reasonable request.

## Abbreviations

g/dl: grams per decilitre
PE: preeclampsia
DMCH: Dhaka Medical College Hospital
LR: Logistic regression
WHO: World Health Organization
SBP: systolic blood pressure
DBP: diastolic blood pressure
BP: blood pressure
BSc: Bachelor of Science
IRB: Institutional Review Board (BRB)
SSC: secondary school certificate
HSC: higher secondary school certificate
BMI: body mass index
Hb: haemoglobin
IRB: Institutional Review Board
LMIC: low and middle income
